# Probiotics in the Management of Lung Diseases

**DOI:** 10.1155/2013/751068

**Published:** 2013-05-08

**Authors:** Esmaeil Mortaz, Ian M. Adcock, Gert Folkerts, Peter J. Barnes, Arjan Paul Vos, Johan Garssen

**Affiliations:** ^1^Division of Pharmacology, Utrecht Institute for Pharmaceutical Sciences, Faculty of Science, Utrecht University, Utrecht, The Netherlands; ^2^Chronic Respiratory Diseases Research Center and National Research Institute of Tuberculosis and Lung Diseases (NRITLD), Department of Immunology, Shahid Beheshti University of Medical Sciences, Tehran, Iran; ^3^Airways Disease Section, National Heart and Lung Institute, Imperial College London, London, UK; ^4^Danone Research Centre for Specialised Nutrition, Wageningen, The Netherlands

## Abstract

The physiology and pathology of the respiratory and gastrointestinal tracts are closely related. This similarity between the two organs may underlie why dysfunction in one organ may induce illness in the other. For example, smoking is a major risk factor for COPD and IBD and increases the risk of developing Crohn's disease. Probiotics have been defined as “live microorganisms which, when administered in adequate amounts, confer health benefits on the host.” In model systems probiotics regulate innate and inflammatory immune responses. Commonly used probiotics include lactic acid bacteria, particularly *Lactobacillus*, *Bifidobacterium*, and *Saccharomyces*, and these are often used as dietary supplements to provide a health benefit in gastrointestinal diseases including infections, inflammatory bowel disease, and colon cancer. In this respect, probiotics probably act as immunomodulatory agents and activators of host defence pathways which suggest that they could influence disease severity and incidence at sites distal to the gut. There is increasing evidence that orally delivered probiotics are able to regulate immune responses in the respiratory system. This review provides an overview of the possible role of probiotics and their mechanisms of action in the prevention and treatment of respiratory diseases.

## 1. Introduction

There is growing evidence as to how diet and nutrition influence the microbiome and interact with the immune system to ultimately improve human health, for example, fecal bacteriotherapy, whereby the microflora of a healthy patient is transplanted to a patient with ulcerative colitis (UC) [1]. This suggests that the composition of the microbiome has an important role in intestinal inflammation and that restoration of a “healthy microbiome” can promote remission of disease. Probiotic bacteria are already used to treat or prevent a wide range of human diseases, conditions, and syndromes including antibiotic-associated diarrhea [2, 3] and inflammatory bowel disease occurring as a consequence of surgical treatment [4]. In addition, they have been proposed to be of potential benefit in increasing numbers of therapeutic areas, particularly in the prevention or treatment of many different chronic inflammatory diseases [5]. Probiotics are characterized as “live organisms, which when applied in adequate amounts confer a health benefit to the host” [6, 7]. Recently, for example, probiotics have been shown to have beneficial effects in models of neuronal inflammation and pain. In particular, probiotics have been reported to decrease the perception of visceral pain induced by distension or inflammation [8, 9].

Emerging studies also indicate that dietary supplementation with probiotics could exert a wide range of beneficial effects on humans with respect to reduced infections and seasonal illness and in the attenuation of the perception of symptoms and disease duration [10–12]. Despite this, many aspects of the probiotic-host immune system crosstalk are still unknown.

Probiotics can exert pleiotropic effects, including a protective role in the intestinal tract where they exert direct antimicrobial effects by competing with local pathogens and indirectly by enhancing intestinal barrier functions [13]. In addition, probiotics have the ability to modulate the host's local and systemic mucosal immune systems [14]. For example, probiotics can change the mucosal immune system towards a noninflammatory, tolerogenic pattern by increasing IL-10 levels [15]. Moreover, the induction of CD4^+^Foxp3^+^ regulatory (T_reg_) cells by probiotics inhibits the production of proinflammatory cytokines and may skew T cells towards a T-helper (Th) 1 phenotype [16–19]. The precise cytokine profile depends upon the nature and strength of the stimulus and the strain of probiotic bacteria that was administered [20, 21]. Moreover, the method of growing and preparing probiotic bacteria for consumption can influence their biological activity. Finally, while probiotics are applied predominantly as living bacteria, bacterial lysates have also been described as having immunomodulatory and anti-inflammatory effects as well (see Table 1).

In this regard, it is important to gain a better understanding of the immunomodulatory mechanisms underlying probiotic functions in order to improve the targeting of probiotic interventions by selecting the optimal strain(s). The fact that probiotic treatment can modulate immune responses in the lung [22] and, in particular, the encouraging indications that microbial stimulation of the gut can enhance the T regulatory response in the airway [23], emphasizes the therapeutic potential as well as the need for greater understanding of the mechanisms underlying effects of specific probiotic strains. In this regard, identification of the key immunoregulatory components of bacteria and/or their metabolism and confirming that results obtained in animal models translate into human models will be critical to the selection of strains and treatment strategies that are most likely to meet with success in preventing or treating human airways diseases such as asthma and COPD.

Most probiotics contain lactic acid-producing bacteria (LABs, e.g., *Lactobacillus*, *Streptococcus*, *Bifidobacterium,* and *Enterococcus sp*.) or nonpathogenic yeasts such as *Saccharomyces boulardii* [24, 25]. These products are advocated for the prevention and treatment of various medical conditions, including gastroenteritis, *Clostridium*-associated diarrhea, inflammatory bowel disease, food allergies, and dental cavities. These applications of LAB have been based on various hypothesized health-promoting attributes including antimutagenic activity [26], anticarcinogenic and -tumor effects [27–30], hypocholesterolemic properties [28], inhibition of intestinal and food-borne pathogens [29], and the promotion of T- and B-cell proliferation [31]. Thus, understanding the immunological mechanism of probiotic action is important in rationalising the impact of these agents in a variety of diseases. Many *in vitro* and *in vivo* studies have been conducted examining the effects of probiotics in disease models. Most *in vitro* studies have examined the effects of probiotics on the production of cytokines from human PBMCs in order to define the basis for their clinical benefit [32, 33]. However, translation of these results into *in vivo* effects in man in randomized controlled clinical trials will require better models of disease and understanding of probiotic function in order to enable the selection of optimal strains and prediction of clinical efficacy.

The microflora hypothesis proposes that antibiotic use and dietary differences in industrialized countries have resulted in perturbations of the gastrointestinal microbiota thereby disrupting the normal microbiota-mediated mechanisms of immunologic tolerance in the mucosa leading to an increase in the incidence of allergic diseases including asthma [34]. This hypothesis provides a rationale for the application of probiotics to aim for normalization of immunological balance and treatment of disease. *Lactobacilli *and *Bifidobacteria* strains are often employed as (potential) probiotics, many of which are useful microorganisms in dairy technology with a long-documented history of use in foods.

## 2. Immunomodulatory Effects of Probiotics

**Figure 1:**
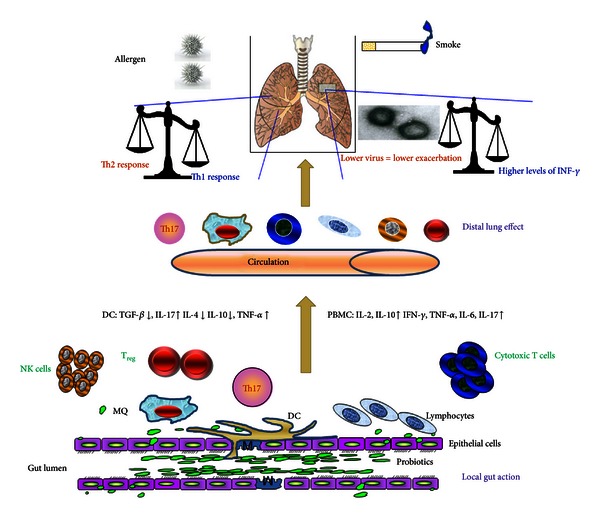
The putative immunomodulatory functions of probiotics on lung disease asthma and COPD. The immune and inflammatory drivers of allergic asthma (left side of figure) and of COPD (right side of figure) may be modified by strain-specific probiotics. The precise mechanisms by which gut-located probiotics can cause immunomodulation in the airway are unclear but may reflect changes in blood and local immune cells including T-cell subsets.

The mechanisms by which probiotic bacteria elicit their effects are not fully understood. The immune response to probiotics is generally thought to be strain dependent, with differences proposed to be due to the diverse protein and glycan/carbohydrate profiles present in their cell walls, the differing CpG content of their DNA, and possibly the metabolites and other molecules they excrete [35]. The beneficial effects of probiotics are thought to be based partly on their ability to differentially regulate the production of anti and proinflammatory cytokines and the balance between types of T cell responses such as Th1/Th2, T_reg_, and Th17 responses [36–38] (see Figure 1).

Probiotic treatment of infectious diarrhea in both adults and children appears efficacious, although evidence of their effectiveness in other diseases often remains to be confirmed in placebo-controlled randomized clinical trials [39–41]. In diarrhea both antipathogenic mechanisms on the microbiota level as well as immunomodulation of the host mechanisms may underlie the clinical benefit of probiotic therapy. At present it is unclear what the relative contribution of these different mechanisms is. Oral administration of *Lactobacilli* may modulate cytokine profiles not only at the intestinal level but also systemically [34]. To date, several LAB strains have been shown to enhance cell-mediated immune responses, including T-lymphocyte proliferation, mononuclear cell phagocytic capacity, and NK cell tumoricidal activity [42] (see Figure 1).

Additional medical applications have been proposed, although their true efficacy will depend upon the outcomes of future experimental and clinical studies. Indeed, despite the widespread advocacy of the clinical benefit of probiotics, evidence-based support for their use is limited and in need of continuing evaluation.

Current ideas center on the hypothesis that administration of probiotic bacteria to the airway mucosa is not required to treat airways disease. Thus, LAB can protect host animals from airway infection through an interaction with GALT such as those in Peyer's Patches in the gut eliciting an indirect enhancement of respiratory immunity [43]. However, it has also been proposed that the protective effects of probiotics (both intranasal and oral) are associated with activation of proinflammatory NK cell and/or macrophages within the airway mucosa [43, 44]. In support of this, Koizumi et al. [45] showed that treating mice with *Lactobacillus pentosus* augmented the activity of NK cells within the spleen and stimulated NK1.1-positive NK and NK T cells to produce IFN-*γ*. The increase in IFN-*γ* production did not occur through direct action of *Lactobacillus pentosus* on NK cells but was dependent on IL-12 produced by CD11c^+^ DCs following a TLR2 and/or TLR4-dependent interaction between the DC and LAB [32, 46]. Strains of LAB differ greatly in their ability to induce high levels of IL-12 in human DCs and consequently DC-dependent IFN-*γ* production by NK cells [47].

The effect of probiotics on other inflammatory cells has also been described. For example, the involvement of Th17 and T_reg_ cells in lung disease has become clear. Th17 cells are a subset of CD4^+^ T cells that produce the proinflammatory cytokine IL-17 [48]. Th17 cells have recently been shown to play a critical role in clearing pathogens during host defense reactions and in inducing tissue inflammation in autoimmune disease [48]. T_reg_  cells are considered to be the master regulators of the immune response in both humans and rodents. Defects in the transcription factor FoxP3, which defines the T_reg_ lineage, result in multiple autoimmune diseases and atopy [49, 50] demonstrating the central role of FoxP3^+^CD4 cells in immune homeostasis.

Karimi et al. have demonstrated that 9 days oral administration of *Lactobacillus reuteri* resulted in a significant increase in the percentage and total number of spleen CD4^+^ CD25^+^ FoxP3^+^ T cells [51]. In addition, increases in FoxP3 mRNA expression in peribronchial lymph nodes has been noted upon administration of *Bifidobacterium lactis Bb12*, *LGG,* and *Lactobacillus casei DN114001* suggesting the induction of T_reg_ cells by these strains [52]. In addition to enhancing T_reg_-cell numbers, LGG has shown effects in a number of Th2-, Th1-, and Th17-mediated disorders [53, 54].

## 3. Probiotics as a Therapeutic Approach in Prevention of Lung Diseases

### 3.1. Asthma and Allergic Reactions

The rise in allergic disease is becoming a major global health issue fast. Whilst this was first evident in the more developed countries of Australasia, Western Europe and North America where more than 40% of the population may be affected at some stage [59, 60], it is now becoming apparent in virtually all regions of the world undergoing industrial development and westernization [61]. International trends, therefore, provide some indication that environmental changes can affect immune function regardless of genetic background. However, recent evidence that non-Caucasian races may be even more susceptible to allergic disease [62, 63] has alarming implications for the most populous areas of the world undergoing rapid urbanization.

Although the exact mechanism(s) behind the antiallergic action of probiotics remain obscure, studies have highlighted several potential components of this response. The development of mucosal and systemic tolerance relies on immunosuppressive mechanisms orchestrated by T_reg_ cells that attenuate both Th1 and Th2 responses, and there is accumulating evidence linking the immunomodulatory function of microbial components and/or commensal bacteria to the induction of T_reg_ cells and their associated cytokines.

Extensive studies examining probiotic modulation of allergic reactions have been reported for atopic eczema with LGG as the probiotic [63–67]. However, clinical trials of probiotics in the treatment of eczema and other allergic diseases have yielded inconsistent results [68–72]. For example, two studies using LGG, with similar study designs, resulted in either a decrease in eczema [73, 74] or no clinical benefit [75].

Despite the inconsistent effects seen in some studies, several allergic diseases may be prevented with treatment of the intestinal flora with probiotics [76, 77] and probiotics have been proposed as being able to both prevent and treat several allergic diseases [78]. In a murine model of asthma, administration of *Lactobacillus reuteri*, LGG, and *Bifidobacterium breve* decreased airway hyperresponsiveness, the number of inflammatory cells in bronchoalveolar lavage (BAL) fluid, and inflammation of lung tissue [58, 79].

In addition to the prevention of experimental asthma, probiotics can prevent atopic dermatitis in infants and alleviate food allergy [80–82]. Recently, Donkor et al. reported that some probiotics induced significant amounts of proinflammatory cytokines, including IL-2, which is a critical cytokine for the clonal expansion of recently antigen-activated T cells and also in T_reg_ homeostasis [32] (see Figure 1).

Specific bacterial strains have been shown to induce IL-10 producing T_reg_ cells *in vitro* [82]. *In vivo*, effects on both regulatory and effector T cells by *Lactobacillus casei DN114001* have been associated with decreased allergic skin inflammation. In addition, *in vivo* treatment with a preparation of heat-killed *Mycobacterium vaccae* can induce allergen-specific T_reg_ (CD4  CD45Rb^lo^ IL-10^+^) cells that confer protection against allergic airway inflammation [83]. Furthermore, Feleszko and colleagues demonstrated that early-life treatment with LGG leads to an attenuated allergic airway response in adult animals that is associated with an increased T cell expression of Foxp3 [84], whereas *Bifidobacterium infantis* induces Foxp3^+^ T cells that protect mice against *Salmonella typhimurium* infection [85].

Interestingly, oral administration of *Enterococcus faecalis FK-23* has recently been shown to suppress the asthmatic response and that this is associated with attenuation of Th17 cell development [86].

Overall, it is unlikely that LABs directly enter into the circulation and then into the lungs. By contrast, they are likely to have an immunomodulatory action affecting the function of immune cells that migrate into the lung. The precise mechanisms underlying the favorable effects of probiotics on the respiratory system are unclear, and several mechanisms have been proposed as a result of *in vitro* and *in vivo* animal experiments. In addition to modulation of the intestinal microbiota, probiotics improve the barrier function of the intestinal mucosa, reducing leakage of antigens through the mucosa and thereby the amount of allergen that the lung may be exposed to via the circulatory route. In addition, direct modulation of the immune system may occur through the induction of anti-inflammatory cytokines or through the increased production of secretory IgA or via activation of T_reg_ cells and skewing of Th1, Th2, and Th17 cell activation or alterations in macrophage function resulting in reduced allergic responses. For example, Salva et al. showed that changes in the BAL cytokine profile occurred after LAB application and that these were associated with an increase in the number and activity of phagocytic cells and with increased levels of allergen-specific antibodies in serum and BAL [55]. 

### 3.2. Viral Infections and Asthma

Respiratory infections, particularly viral infections, directly affect morbidity and mortality and are indirectly a contributing factor not only to asthma exacerbations but also to the development of the disease [87]. It has been speculated that identifying probiotic organisms capable of reducing viral infections in early life, or in utero, may prevent the development of asthma [88]. As indicated above, emerging evidence supports the role of probiotics, for example, LGG and *Lactobacillus casei* strains *Shirota* (*LcS*) and *DN114001*, in the prevention and/or treatment of bacterial and viral infections in the gastrointestinal and respiratory systems including influenza [57, 89–93].

Respiratory pathogens are recognized by pattern recognition receptors (PRRs). For example, Toll-like receptors (TLRs), nucleotide-binding oligomerization domain (NOD-) like receptors, transcription factors, cytokines, and chemokines play essential roles in sensing perturbations in the health of the lungs. The expression of many PRRs is high in lung inflammatory cells such as neutrophils, monocytes, macrophages, and epithelial cells, and they are able to respond rapidly to increased levels of PAMPs and DAMPs which are associated with exacerbation of lung diseases. It is possible, therefore, that modulation of these responses induced by LAB may have an impact on immune cell activation in the lung.

In addition to oral delivery of probiotics, probiotic efficacy has been demonstrated following nasal challenge. Thus, Harata et al. reported that intranasal administration of LGG could protect mice from H1N1 influenza virus infection by regulating respiratory immune responses [94]. In addition, probiotic use has been associated with lower incidence of ventilator-associated pneumonia [95], reduced respiratory infections in healthy and hospitalized children [96, 97], and reduced duration of infection with the common cold [98].

### 3.3. Chronic Obstructive Pulmonary Disease (COPD)

COPD is currently the 4th biggest killer worldwide and is expected to be the third leading cause of death over the next 10 years [99]. Smoking is the most important lifestyle risk factor for pathogenesis of COPD [100] and lung cancer [101]. COPD and IBD share many epidemiological and clinical characteristics, as well as inflammatory pathologies [102–107]. The crosstalk between the pulmonary and intestinal mucosal in chronic inflammatory diseases such as COPD and IBD has recently been extensively reviewed [108].

The level of inflammation in the COPD airways is a strong correlate of disease severity in patients and is critically involved in disease development experimentally [109–111]. Attention has traditionally centered on the roles of macrophages and neutrophils in disease development [112]. However, lymphocytes have received increasing attention, and reports from many laboratories have provided insight into functions of T cells and NK cells in the pathogenesis of COPD [113–115].

COPD has been characterized by exacerbations of symptoms including increased dyspnea, enhanced sputum, enhanced inflammation, and decline in lung function [116, 117]. COPD exacerbations are psychologically destructive, since patients with exacerbations have a lower quality of life and decreased mobility, leading to increased depression [118]. 40%–60% of exacerbations in COPD are caused by viral infections [119, 120], and importantly, in animal models viral infection following cigarette smoke exposure leads to enhanced pulmonary inflammation, increased alveolar apoptosis, accelerated emphysema, and airway fibrosis [120]. However, the underlying cellular mechanisms responsible for these effects remain unresolved. 

It is generally accepted that the early immune response following viral infection relies on the recognition of viral PAMPs through the Toll-like receptors (TLRs) mainly TLR3, TLR7, and TLR9 [121, 122]. Stimulation of these receptors found on DCs and other inflammatory cells leads to the activation of NK cells through production of type I IFNs, IL-12, IL-18, and IL-15 [123–126]. NK cell activation is critically important to the early control of viral infections and occurs several hours to a few days following infection [127]. NK cells were originally thought of solely as killer cells due to their ability to directly destroy virus-infected cells [128]. More recently, however, attention has been given to the noncytotoxic functions of NK cells [129]. Activated NK cells produce large amounts of IFN-*γ* [130] and NK-cell-derived IFN-*γ* is critical to the inflammatory processes that control viral infections [131–133] (see Figure 1). Thus, NK cells along with their release of mediators are considered as important cells regulating the inflammatory response that occurs during COPD exacerbations. 

Cigarette smoking impairs human NK-cell cytotoxic activity and cytokine release [134]. NK-cell activity is lower in smokers compared to nonsmokers [11] but daily intake of *LcS* increases natural killer cell activity in smokers [135]. This suggests that probiotics may be useful in COPD patients, particularly those with frequent viral infections [11]. Of interest, a diet supplemented with *Lactobacillus plantarum* prevents cardiovascular disease in smokers [11].

## 4. Future Studies on the Effects of Probiotics in Lung Disease

Recent studies suggest that commensal microorganisms are not identical in their ability to affect host physiology. Ongoing studies aimed at establishing a correlation between the presence of particular microbes and specific diseases will provide much needed insights into their relationships with the immune system. The effect of microbial influences on the immune system and specific cell types has been extensively reviewed [136, 137]. While considerable progress has been made in large-scale analysis of gut commensal microbiota and its effect on the balance of pro- and anti-inflammatory forces of the immune system, many questions remain to be solved including which immune cells are the important targets for probiotic actions. In the future, it will be important to determine the mechanisms behind the probiotic action on the respiratory tract in diseased states. This will enable a rational approach for the use of probiotics in lung disease. A greater knowledge of the intestinal microbiota seen in patients with pulmonary disorders such as allergic diseases and in healthy infants will present opportunities to select more effective strains or combinations of strains to modulate the immune response and treat disease. Because probiotics modulate the composition and/or activity of the intestinal microbiota, it is important to obtain information on the intestinal microbiota culture, not only from fecal samples as is commonly performed but also from those of the associated mucosa.

## Figures and Tables

**Table 1:** Dietary supplementation with lactobacilli that have shown enhanced immune response and protection against respiratory tract pathogen challenge.

LAB treatments	Immune response	Authors
The immune stimulation induced by *L. rhamnosus * CRL1505 (Lr05) and *L. rhamnosus* CRL1506 (Lr06) on the resistance to infection with an intestinal pathogen (*Salmonella typhimurium*) and a respiratory pathogen (*Streptococcus pneumoniae*)	Both strains were able to improve resistance against the intestinal pathogen. Only Lr05 was able to induce a significant decrease in the number of *S. pneumoniae* in the lung, prevent its dissemination into the blood, and induce a significant increase in Th1 (INF-*γ*) and Th2 (IL-6, IL-4 and IL-10) cytokine levels in the bronchoalveolar lavages (BAL)	Salva et al. [55]
2 days before feeding of *L. casei* prior to pathogen challenge	Increased rate ofclearance of *P. aeruginosa* from the lungs increased phagocytic activity of alveolar macrophages, and increased levels of IgA in BAL fluid	Alvarez et al. [56]
Prefeeding of *L. casei* (Shirota strain) for 4 months prior to challenge	Reduced viral titre in nasal washings; increased NK activity of splenocytes and nasal tract mononuclear cells; increased IFNQ and TNFK production by mitogen-stimulated nasal lymphocytes	Hori et al. [57]
OVA-sensitized mice were orally administered with *Bifidobacterium breve* M-16V, *B. infantis* NumRes251, *B. animalis* NumRes252 and NumRes253, *Lactobacillus plantarum* waNumRes8, and *L. rhamnosus* NumRes6. After challenge by OVA inhalation in the lungs, the response to methacholine was measured Pulmonary inflammation assessed by analyzing BALF for the presence of inflammatory cells and mediators	Of the panel of 6 strains, *B. breve* M-16V and *L. plantarum* NumRes8 inhibited (1) the response to methacholine, (2) reduced the number of eosinophils in the bronchoalveolar lavage fluid, and (3) reduced both OVA-specific IgE and (4) OVA-specific IgG1, whereas the other strains did not affect all these parameters simultaneously. *B. breve* M-16V but not *L. plantarum* NumRes8 reduced interleukin 4, interleukin 5, and interleukin 10 Furthermore, *B. breve* M-16V but not *L. plantarum* NumRes8 reduced acute allergic skin reactions to OVA	Hougee et al. [58]

## Abbreviations

APC: Antigen presenting cells
AHR: Airway hyper responsiveness
DCs: Dendritic cells
GALT: Gut-associated lymphoid tissues
FoxP3: Forkhead box protein 3
IL: Interleukin
IF: Influenza virus
IFN-*γ*: Interferon gamma
LGG: *Lactobacillus rhamnosus* GG
LcS: *Lactobacillus casei Shirota*
LAB: Lactic acid bacteria
M: M cells of intestinal epithelium
NK: Natural killer cells
PBMC: Peripheral blood mononuclear cells
PAMPs: Pathogen-associated molecular patterns
Th: T-helper cells
TGF: Transforming growth factor
TNF: Tumour necrosis factor
TLR2: Toll-like receptor 2
T_reg_: T regulatory cells.
